# Moving with Ease: Feldenkrais Method Classes for People with Osteoarthritis

**DOI:** 10.1155/2013/479142

**Published:** 2013-09-03

**Authors:** Robert Webb, Luis Eduardo Cofré Lizama, Mary P. Galea

**Affiliations:** ^1^Merri Community Health Services, Coburg, VIC 3058, Australia; ^2^MOVE Research Institute Amsterdam, 1081 BT Amsterdam, The Netherlands; ^3^Department of Medicine (Royal Melbourne Hospital), The University of Melbourne, Parkville, VIC 3052, Australia

## Abstract

*Objective*. To investigate the effects of Feldenkrais Method classes on gait, balance, function, and pain in people with osteoarthritis. *Design*. Prospective study with pre-/postmeasures. *Setting*. Community. *Participants*. Convenience sample of 15 community-dwelling adults with osteoarthritis (mean age 67 years) attending Feldenkrais Method classes. *Intervention*. Series of Feldenkrais Method classes, two classes/week for 30 weeks. Main outcome measures: Western Ontario and McMaster Universities osteoarthritis scale, Human Activity Profile, stair climbing test, 6-minute walk test, timed up-and-go test, Four Square Step Test (4SST), gait analysis, and assessment of quality of life (AQoL). *Results*. Participants improved on the 4SST and on some gait parameters. They also reported a greater ease of movement. *Conclusions*. A 30-week series of Feldenkrais classes held twice per week was feasible in the community setting. The lessons led to improvements in performance of the four square step test and changes in gait.

## 1. Introduction

Osteoarthritis is a disabling and costly disease in Australia, with almost 50% of people aged over 65 years having arthritis [1]. It is characterized by progressive degeneration of articular cartilage, resulting in a reduction of joint space, with accompanying pain, stiffness, and increased difficulty in moving. The knee joint is most frequently affected, followed by the hip, resulting in impaired walking and difficulty in performing daily tasks [2]. There is no cure for OA or interventions that delay disease progression. Current management strategies include education, exercise, and pharmaceutical interventions. However, medications result in only short-term benefits and have significant adverse effects [3]. A nondrug intervention such as exercise is desirable as it is safe and effective and improves muscle strength and joint stability [4].

Atrophy of muscles controlling the hip and knee and reduced ankle strength have been observed in those with OA, thus altering the biomechanics of movements such as walking [5]. Ankle plantar flexion power is important for proper walking function and is a strong predictor of walking speed [6, 7]. However, significant reductions of ankle power have been observed with aging when walking at same speeds than young subjects, which increases the reliance upon hip and knee muscles [8, 9]. These walking patterns are more exacerbated in older adults with poor physical performance [10] and may contribute to further joint deterioration [11]. Many older people with OA have more than one joint affected [12]; therefore treatment needs to address the entire movement pattern. 

The Feldenkrais Method has the potential to help older people with OA. Developed by Dr. Moshe Feldenkrais, the method is a gentle form of exercise which has been shown to be acceptable for older people who have limited movement [13]. The Feldenkrais Method is taught in two parallel forms, Awareness Through Movement (conducted as a group exercise) and Functional Integration (one-on one approach). This study explores the effectiveness of Awareness Through Movement lessons in helping older people with OA.

Awareness Through Movement lessons are verbally guided explorations of movement that are about 30–60 minutes long. Each lesson explores movement related to a particular function (e.g., walking) to enhance awareness of how movements are performed and invite the participant to investigate how they might expand their action and ability to function. The lessons address habitual patterns of movement and expand a person's self-image. By exploring novel movement sequences, attention is drawn to parts of the self which the person may not be aware of and may have excluded from their functioning. The method aims for a heightened self-awareness, an expansion of a person's repertoire of movement, and improved functioning where the whole body cooperates in movement and where maximum efficiency is achieved with minimum effort. Dr. Feldenkrais described the aim of the method as “a person who is organised to move with minimum effort and maximum efficiency, not through muscular effort, but through increased consciousness of how movement works” [13].

The Moving With Ease program is a selection of Awareness Through Movement lessons from the Feldenkrais Method. Because the lessons are gentle and enjoyable, they may enable people with OA to move more easily and better manage their pain. The self-exploratory nature of the classes provides an opportunity for participants to become aware of how they move, thus learning to minimize their functional limitations. Therefore the lessons become a form of self-management that addresses a significant aspect of the process of disablement in people with osteoarthritis [11].

Several recent studies have demonstrated the effectiveness of the Feldenkrais Method in improving balance related outcomes for older people [14–16]. To date, no studies have investigated the feasibility or effectiveness of Awareness Through Movement lessons for people with OA. 

The purpose of this study was to investigate whether community-dwelling adults with osteoarthritis undertaking a series of Feldenkrais Method classes improved on measures of mobility, function, balance, quality of life, and pain. This was a pragmatic study of a group of older adults with osteoarthritis already enrolled in Feldenkrais Method classes.

## 2. Methods

The project was approved by the Human Research Ethics Committee at the University of Melbourne.

### 2.1. Participants

A sample of convenience was recruited, drawn from community-dwelling older adults with osteoarthritis responding to an advertisement of Feldenkrais Method classes to be conducted in a community health setting. 

Inclusion criteria were aged between 55 and 75 years, OA diagnosed by a medical practitioner using the clinical criteria for diagnosis of OA of the hip and/or by radiographs [17], and able to rise from the floor, walk for 6 minutes and manage their pain. Those with previous joint replacements, who were wait-listed for lower limb joint replacements, or who had rheumatoid or other inflammatory arthritis, or major neurological conditions, were excluded. All participants provided informed consent. Those currently receiving any additional intervention related to mobility were excluded from the study. 

### 2.2. Procedures

Participants were assessed on outcome measures prior to starting the classes and at completion of the program. Assessments included the timed up-and-go test (physical function) [18], the Four Square Step Test (dynamic balance) [19], stair climbing test (leg power) [20], 6-minute walk test (endurance), assessment of quality of life (AQoL) (illness, independence, social relationships, physical senses and psychological well being) [21], Western Ontario and McMaster Universities osteoarthritis index (WOMAC), a disease-specific measure of health status (pain, stiffness and function) in OA sufferers [22], and the Human Activity Profile (HAP) (type and extent of physical activity) [23]. Halfway through and on completion of the program, participants were requested to complete questionnaires for the WOMAC, AQoL, and HAP. Participants were also asked to complete a questionnaire about their experience of the classes at the end of the intervention period.

Physical assessments were performed in the Movement Laboratory at the Rehabilitation Sciences Research Centre by independent assessors. For gait analysis, reflective markers were attached according to the Vicon Plug-in Gait model. Participants completed several walking trials on a level walkway at self-selected speed and at 1.2 m·s^−1^ and 1.4 m·s^−1^. An eight-camera motion measurement system (VICON) and 3 AMTI force plates (Watertown), were used to collect kinematic and kinetic data at the ankle, knee, and hip. 

### 2.3. Intervention

The Moving With Ease program comprised a series of 60 Awareness Through Movement lessons drawn from the vast catalogue of lessons which comprise the Feldenkrais Method (see Supplementary Material available online at http://dx.doi.org/10.1155/2013/479142 for a brief description of each lesson). The lessons were delivered by the Feldenkrais practitioner who devised the program (RW). Classes were conducted for one hour, twice weekly, in three segments of ten weeks each, with a short break between segments. Each lesson was recorded and made available to those who had missed a lesson, with a complete set of the lessons provided to participants at the end of the program.

The lessons were selected with the aim of improving hip, knee, and ankle function in the context of improving overall function. Each 10-week segment had an overall theme. The first segment focussed on helping participants to learn to pay attention and develop awareness, learning self-care, and improving fundamental range of motion (flexion, extension, and rotation). Segment 2 focussed on the function of the pelvis and lower limbs. The themes included gaining control of the pelvis, freeing the hip joints, and improving ankle, knee, and hip function. Segment 3 focussed on improving balance, improving walking, and integrating ankle, knee, and hip function with walking. Each individual lesson had a functional theme. Lessons would often return to previous functional themes building upon them as the program progressed. An example of this thematic development can be seen on video on *Youtube* (http://youtu.be/V9tf21itKuE).

## 3. Results

**Figure 1:**
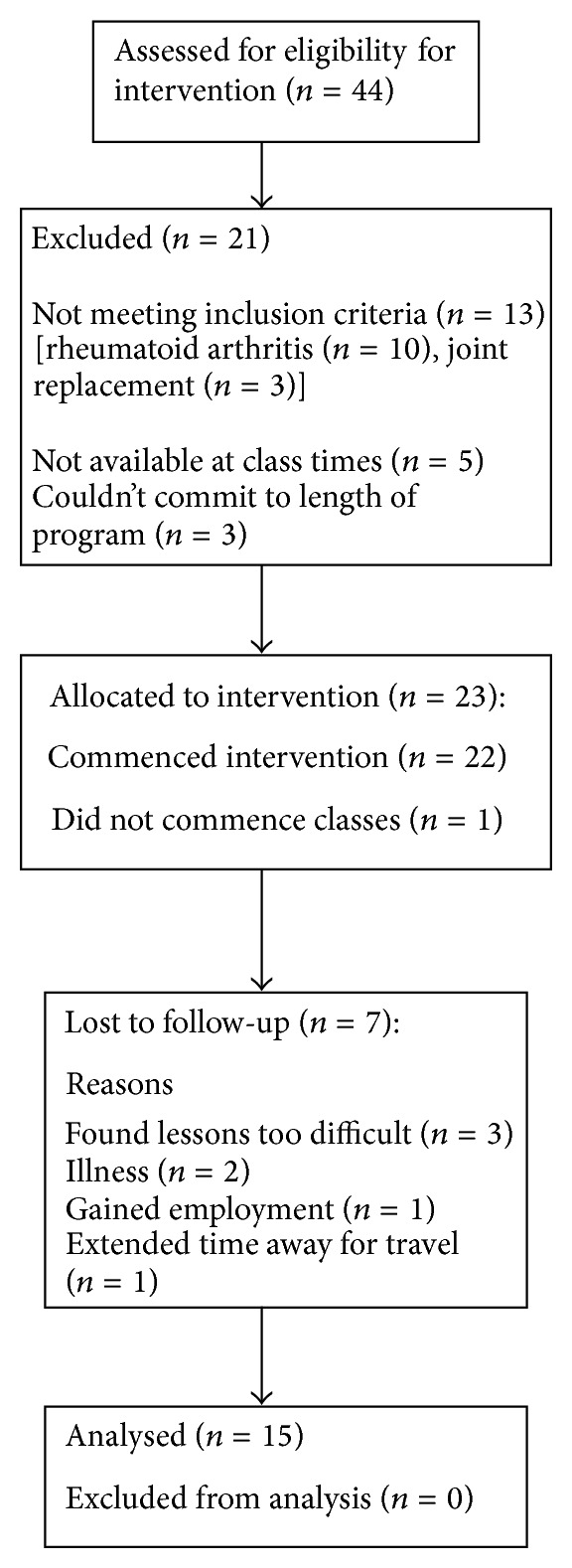
Flow chart of participant recruitment and retention.

Figure 1 shows recruitment and retention of participants in the study, and Table 1 shows participant characteristics. Of the original sample of 23 participants, 15 participants returned for retesting after 12 months. Some questionnaires were incomplete.

### 3.1. Clinical Tests and Questionnaires

Given the small sample size, statistical analyses were not conducted on the results of the clinical tests and questionnaires, therefore the raw scores have been provided in Tables 2, 3, 4, and 5. On the HAP and the WOMAC, which were completed on three occasions, there appeared to be an overall decline in function at the 6 month time point, with an improvement at the final assessment. There was no observable trend in changes on the clinical tests except for a uniformly positive improvement on the Four Square Step Test (Table 5).

### 3.2. Gait

**Figure 2:**
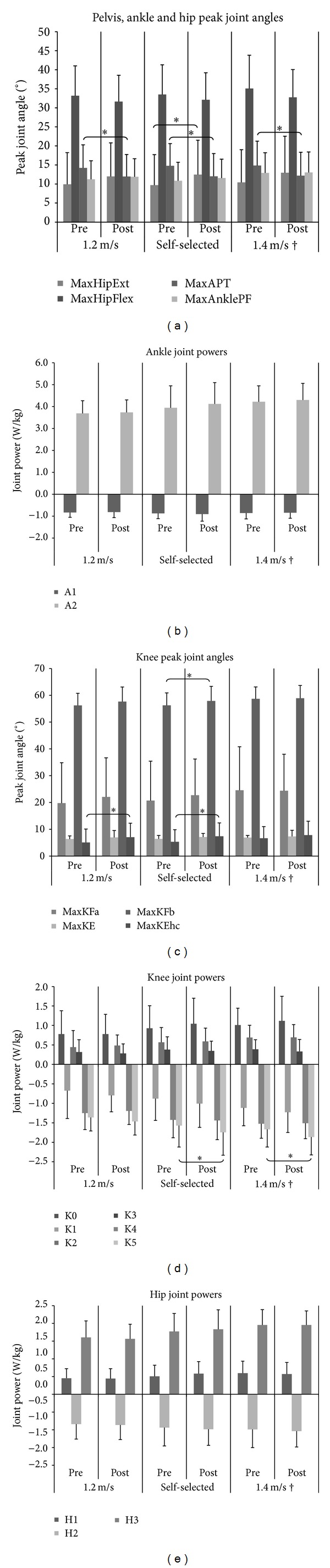
Peak joint angles and joint powers.

All participants were able to walk without external aids; however, two of them were not able to walk at the highest speed (1.4 m·s^−1^) and were therefore excluded from all analyses of this condition. Both subjects reported left knee OA and one of them also had OA in the left ankle and right toes. Descriptive statistics and repeated measures ANOVAs for the spatiotemporal and kinetic measures are summarized in Tables 6 and 7.  Figure 2 shows bar graphs depicting average peak joint angles (±SD) and peak joint powers (±SD). An asterisk highlights significant differences after intervention. 

#### 3.2.1. Spatiotemporal Measures (Table 6)

Spatiotemporal measures of cadence, step length, stride length, step width, percentage of gait cycle in stance, and percentage of gait cycle in single stance were compared before and after intervention. When comparing the effect of intervention for all subjects at all speeds conditions, no significant differences were found for any measures (*P* > 0.01). No differences were found for the same measures when participants were grouped according to joints affected. 

#### 3.2.2. Kinematic Measures (Table 6)

When comparing pre- and post-interventions peak joint angles for all subjects at all speeds tested, no significant differences were found for most of the measures (*P* > 0.01). However, significant increases were found for hip extension during late stance (2.8° increase), maximal knee flexion during swing (1.6° increase), and knee extension at heel contact (2.1° increase) during walking at self-selected speed (*P* < 0.01). Knee extension at heel contact was also found to be significantly higher (2.0° increase) after intervention when participants walked at 1.2 m·s^−1^. Interestingly, maximal anterior pelvic tilt was found to be significantly reduced after intervention at all speeds (*P* < 0.01), with an average reduction of 2.6°.

#### 3.2.3. Kinetic Measures (Table 7)

No significant increases were found for ankle peak joint power generation (A2) at all speed conditions after intervention. Except at self-selected speed, where subjects walked faster than prior to intervention, ankle absorption power (A1) remained similar after intervention. 

At the knee, most of the intervention effects were observed when subjects walked at self-selected and 1.4 m·s^−1^ conditions, but not at 1.2 m·s^−1^. At the two highest speed conditions, no significant increases were found for knee power generation at the beginning of stance (K0) and decreases for knee power absorption (K1). Most importantly, a significant increase (*P* < 0.01) in knee power absorption at the end of the gait cycle (K5) was found at the same conditions with an average increase of 0.2 W/kg after intervention.

At the hip, no significant changes were observed at all speed conditions for all peak joint powers analysed. Small increases, however, were observed for hip power generation after toe-off (H3) in the post-intervention gait pattern, especially at self-selected speed (0.06 W/kg increase).

### 3.3. Participant Comments

Class attendance was high (76.5%), and feedback from the satisfaction survey was positive. All 15 participants said they enjoyed the program “very much.” Eleven of the fifteen participants reported improvements in their ability to do everyday things since the beginning of the program, including going up and down stairs, ability to stay longer in the garden, better deportment, improved walking, and more flexibility. When asked to describe what they had learnt by participating in the program, comments included “how exercise/movement is crucial to managing pain,” “to exercise where it is comfortable, not to force it,” “to walk with a more fluid, gentle motion,” and “learnt to incorporate some of the exercises into my daily life.” Participants were asked to comment on their experience of pain and, in particular, the pain associated with their osteoarthritis after participating in the program. Ten of the fifteen participants said their pain level had improved, three were unsure and two said they had not noticed any difference. Comments included “the pain is continual, but I manage it better,” “at the end of the session I was free from pain and felt energized,” “I can experience less pain in the knees, which is where the osteo appears for my body,” “the lessons…eased the pain in my lower back,” “no pain in the knees when going up stairs,” and “it is not a cure! however, it is the best “exercise” I have experienced for managing my osteoarthritis”. Participants were asked to comment on their experience of the program in relation to balance, confidence, or walking. Eight of the fifteen participants reported an improvement in one or more of these areas. Comments included “my balance and confidence in my walking have all improved,” “feel more confident of walking/climbing up/down,” “less pressure on the knees when walking,” “getting up and down from the floor is much easier” and “the program has helped me in every way. The best thing about it is that I know I can do this exercise”. Participants were asked whether they experienced any other benefits from attending the program. Comments included, “It has made me move in time with my body,” “I feel more energetic, brighter, sleeping better,” “I am more positive even though pain is still prevalent,” and “enabling, empowering … . I feel so confident and grateful that I have found an exercise that suits me.” When asked whether participants would undertake Feldenkrais classes in the future, eight responded “definitely,” three responded “probably,” three responded “maybe, depending on cost,” and one person responded “no”. 

## 4. Discussion

Given the participants' comments on the final questionnaire, it is clear that the physical assessments and questionnaires did not adequately capture the types of functional changes resulting from undertaking the Feldenkrais classes. These included a change in the quality of their movement (i.e., moving with ease), ability to manage their pain, the ability to get up and down from the floor and climb stairs, better balance, and improvement in walking. The small sample size makes it difficult to conclude that there were positive changes in function; however, the uniformly positive improvement on the four square step test, coupled with the changes in gait detailed below indicating a more upright posture, is suggestive of an improvement in balance. Since people with OA have balance deficits [24], an improvement in balance for this group would be an important outcome. 

We undertook gait analysis in order to identify any changes in gait patterns associated with the Feldenkrais classes. To our knowledge, this is the first study to do so. The findings of decreased anterior pelvic tilt across all speed conditions may indicate an effect of the Feldenkrais intervention in correcting upright posture. This kinematic change may have reduced the forward inclination of the trunk and reduced loads at the low back when walking [25]. At least 15 of the Feldenkrais lessons were focussed on activation of the abdominal muscles in combination with other movements. It is possible that this focus led to increased muscle activity or strength in the abdominal muscles which, in turn, contributed to the decrease in anterior pelvic tilt. This reduction may be beneficial especially for those subjects suffering from OA in the lumbar spine (6 participants). Increases in anterior pelvic tilt have been also reported to be significant in patients with severe OA [26]. Therefore, it may be possible that reductions in this kinematic measure after intervention may be, in itself, a reflection of gait changes that may contribute to decelerate OA severity progression. In addition, reductions in anterior pelvic tilt may contribute to reducing the probability of falls and reduce energy cost when walking [27, 28]. 

Anterior pelvic tilt reductions coupled to an increased hip extension may allow increases in hip extension (absorption) power (H2) leading to higher elastic energy storage mainly in the iliopsoas muscle [29, 30]. This energy is released later in period between maximal hip extension in the stance phase and maximal hip flexion in the swing phase during the second propulsive hip flexion power (H3). It is possible that stretching of the rectus femoris, due to increased hip extension and reduced anterior pelvic tilt, may have also contributed to H3 through the release of elastic energy during late stance [31]. Nonetheless, a reduction in H3 associated with hip flexion reduction was found at the two controlled speed conditions. 

DeVita and Hortobagyi [32] proposed that normal aging produces a shift in the locus of function when walking, with an increase in proximal muscle activity and a reduction in distal muscle activity to propel the body forward. Considering that participants in this study increased their self-selected speed and were able to walk at the controlled speed conditions, it was expected that there would be a restoration of the distribution of muscle activity across joints. However, no significant increases in A2 power and ankle plantar flexion were found. Alternatively it is possible that strategies in the frontal and transverse planes, not measured in this gait analysis, may have increased their contribution to propulsion. A large proportion of the Feldenkrais lessons involved rotational movements of the spine. Also, much of the Feldenkrais program was aimed at improving walking by bringing into awareness the role of pelvic rotation, the role of the counter rotation of the thorax and shoulders, and the role of the head in walking. Improvement in spinal rotation and the emphasis on the involvement of the upper body in walking, coupled with a straighter posture, may have led to a better ability to rotate the pelvis and hip when the leg is in stance [25]. This may also partly explain why there are reductions in H3 and K5 and greater flexion of knee during swing without affecting step/stride lengths and gait speed. 

A significantly lower K5 (higher absorption) at the end of the gait cycle indicates an increased eccentric activity of the hamstring muscles after intervention. This may have influenced the higher knee flexion observed at heel contact at the beginning of a new gait cycle. An increase in K5 power absorption is associated with a reduced step length; however, it may also increase stability of the knee and reduce forward foot speed in order to prepare for landing, leading to reduced slip-induced falls [33, 34]. Nevertheless, our findings showed that step and stride length were both maintained. An increase in hip extension is also used as a mechanism to increase step length [35]. We found increases in this measure which may indicate a change in strategy to maintain step/stride length and make initiation of the new gait cycle more stable. 

Increases in the second peak of knee flexion, significant at the self-selected speed, may contribute to a better foot clearance, decreasing the probability of tripping and falling when the limb is in the swing phase [36]. This knee flexion increase may arise from an increased activity of the hamstring muscles during swing, which is also responsible for an increased K5 at the end of this phase. The Feldenkrais program did include specific lessons targeting the hamstring muscles which may have contributed to increased activity in these muscles. It is possible that an overall increase in muscle activity in the hamstrings after intervention may have not only contributed to reduction in anterior pelvic tilt but also increased their absorptive function at the knee during late swing. 

Only sagittal plane gait analysis was performed in this study. Despite controlling over-ground gait speed using timing gates, significant differences for speed were found when participants were asked to walk at 1.2 m/s. This may be explained by the fact that participants walked closer to the lower speed limit allowed during the baseline assessment and closer to the higher limit during the final assessment. This is also reflected in the higher self-selected walking speed exhibited after intervention for this condition. 

It is a tenet of the Feldenkrais Method that efficient movement occurs when the work is spread throughout the body. Although our analyses have focused on changes in gait, the intention of the lessons was not to focus solely on the lower limbs, but to teach a comprehensive program that would improve overall movement organisation. Future studies could evaluate this aspect further.

## 5. Study Limitations

A major limitation of this study was the lack of a comparison group due to the pragmatic nature of this study. The participant group was a sample of convenience, recruited from people who responded to an advertisement of Feldenkrais Method classes. It is acknowledged that the group was not homogeneous with respect to gender and the joints affected by osteoarthritis. The greater proportion of women in our group reflects the fact that more women are affected by arthritis than men [1] and that, in our experience, women are more likely to volunteer for such projects than men. 

However there were no adverse effects such as falls or reports of injuries during the classes and participants who continued with the program reported meaningful changes in their function. 

## 6. Conclusion

The results, high class attendance (76.5%), and survey feedback indicate that a 30-week series of Feldenkrais classes held twice per week was feasible in the community setting and may be acceptable for other people with OA. The lessons led to improvements in performance of the Four Square Step Test and changes in gait. Further investigation of the Feldenkrais Method for people with OA is warranted.

## Supplementary Material

The Supplementary Material includes a list of all the Feldenkrais Awareness Through Movement lessons undertaken by participants in this study, together with a description of the lessons.

## Figures and Tables

**Table 1:** Participant characteristics.

Participant	Gender	Age	Condition
FP002	M	69	R hip, lumbar spine
FP003	F	73	Lumbar spine
FP004	F	61	R knee
FP006	F	70	Both knees, lumbar spine
FP007	F	72	R knee, lumbar spine
FP008	F	63	Both knees
FP009	F	71	L ankle, L knee, R toe
FP010	F	75	R hip
FP011	F	59	L knee
FP012	F	62	Multiple joint arthritis; fusion of large toes of both feet July 2011
FP013	F	63	L knee
FP017	M	68	Knees, lumbar spine; at reassessment: Morton's neuroma and plantar fasciitis on L; injured cartilage L knee
FP018	F	61	R knee and hip
FP019	F	67	Neck, lumbar spine, knees
FP023	F	72	Both knees

**Table 2:** Human activity profile.

Participant	MAS 1	MAS 2	Change MAS (2 − 1)	MAS 3	Change MAS (3 − 1)	AAS 1	AAS 2	Change AAS (2 − 1)	AAS 3	Change AAS (3 − 1)
FP002	72	64	−**8**	73	**1**	63	41	−**22**	64	**1**
FP003	78	69	−**9**	78	**0**	65	58	−**7**	62	−**3**
FP004	82	65	−**17**	82	**0**	70	40	−**30**	62	−**8**
FP006	82	63	−**19**	82	**0**	68	34	−**34**	64	−**4**
FP007	77	73	−**4**	78	**1**	73	63	−**10**	75	**2**
FP008	70	66	−**4**	66	−**4**	61	48	−**13**	56	−**5**
FP009	57	32	−**25**	53	−**4**	47	4	−**43**	38	−**9**
FP010	75	55	−**20**	70	−**5**	63	30	−**33**	61	−**2**
FP011	82	62	−**20**	82	**0**	71	53	−**18**	—	—
FP012	—	—	—	58	—	—	—		26	—
FP013	70	60	−**10**	70	**0**	61	44	−**17**	63	**2**
FP017	82	76	−**6**	82	**0**	75	59	−**16**	74	−**1**
FP018	80	46	−**34**	61	−**19**	61	8	−**53**	52	−**9**
FP019	82	56	−**26**	78	−**4**	70	47	−**23**	74	**4**
FP023	77	75	−**2**	82	**5**	74	66	−**8**	77	**4**

1 = baseline; 2 = 6 month mark; 3 = final.

MAS (maximum activity score): highest oxygen-demanding activity still being performed; best estimate of highest level of energy expenditure in comparison with peers of same age and gender.

AAS (adjusted activity score): a measure of usual daily activities; best estimate of average level of energy expenditure in comparison with peers of same age and gender.

**Table 3:** WOMAC average scores.

	Pain 1	Pain 2	Change pain (2 − 1)	Pain 3	Change Pain (3 − 1)	Stiff 1	Stiff 2	Change Stiff (2 − 1)	Stiff 3	Change Stiff (3 − 1)	Func 1	Func 2	Change Func (2 − 1)	Func 3	Change Func (3 − 1)
FP002	11.9	13.7	**1.8**	9.6	**−2.3**	3.8	7	**3.2**	10.9	**7.1**	18.1	15.5	**−2.6**	19.5	**1.4**
FP003	4.1	2.4	**−1.7**	6.3	**2.2**	3.3	2.2	**−1.1**	7.9	**4.6**	9.6	8.9	**−0.7**	15.9	**6.3**
FP004	22.9	22.6	**−0.3**	29.4	**6.5**	8.2	9.4	**1.2**	8.4	**0.2**	61.1	66.1	**5**	83	**21.9**
FP006	9	8.4	**−1.4**	24.7	**15.7**	8.1	7.9	**−0.2**	8	**−0.1**	24.2	10.6	**−13.6**	—	—
FP007	5.1	3.2	**−1.9**	10.3	**5.2**	9.3	2.9	**−6.4**	8.1	**−1.2**	29.2	14.5	**−14.7**	22.2	**−7**
FP008	10.8	10.3	**−0.5**	12.8	**2**	11	9.1	**−1.9**	15	**4**	68.2	54.4	**−13.6**	75.2	**7**
FP009	3.9	22.5	**19**	19.2	**15.3**	0.5	9.1	**8.6**	10.7	**10.2**	28.3	49.7	**11.4**	—	—
FP010	9	11.1	**2.1**	4.9	**−4.1**	8.1	7	**−1.1**	4.6	**−3.5**	30.4	33.1	**1.7**	33.4	**3**
FP011	6.2	6.7	**0.5**	6.6	**0.4**	13.3	4.1	**−9.2**	11.5	**−1.8**	33.4	21.6	**−11.8**	42.1	**8.7**
FP012	24.5	—	—	9.2	**−15.3**	14.4	0	—	—		62.9	0	—	—	—
FP013	0	6.7	**6.7**	2	**2**	4.8	4.5	**−0.3**	3.6	**−1.2**	22	18.3	**−3.7**	9.3	**−12.7**
FP017	9.9	12.8	**2.9**	10.5	**0.6**	2.2	7.1	**4.9**	5.5	**3.3**	14.8	25.8	**11**	45.1	**30.3**
FP018	4	12.7	**8.7**	5.9	**1.9**	4.1	10.2	**6.1**	2.8	**−1.3**	12.8	21	**8.2**	37.6	**24.8**
FP019	12.9	5.2	**−7.7**	11.5	**−1.4**	10.7	3.9	**−6.8**	5.7	**−5**	42.6	17.9	**−28.7**	37.4	**−5.2**
FP023	9.8	7.3	**−2.5**	10.7	**0.9**	5	7.1	**2.1**	6.2	**1.2**	27.7	21.2	**−6.5**	20	**−7.7**

Visual analogue version used; 1 = baseline; 2 = 6 months mark; 3 = final. Negative numbers indicate improvement.

Stiff: stiffness; Func: function.

**Table 4:** Assessment of quality of life (AQoL).

Participant	AQoL 1	AQoL 2	Change (2 − 1)	AQoL 3	Change (3 − 1)
FP002	3	4	**1**	4	**1**
FP003	7	7	**0**	5	−**2**
FP004	8	7	−**1**	8	**0**
FP006	9	8	−**1**	6	−**3**
FP007	4	6	**2**	9	**5**
FP008	10	8	−**2**	16	**6**
FP009	22	20	−**2**	10	−**12**
FP010	11	11	**0**	6	−**5**
FP011	7	7	**0**	7	**0**
FP012	11	0	−**11**	—	—
FP013	3	4	**1**	—	—
FP017	7	0	−**7**	1	−**6**
FP018	2	0	−**2**	2	**0**
FP019	6	5	−**1**	6	**0**
FP023	4	3	−**1**	4	**0**

Higher scores mean lower quality of life.

1 = baseline; 2 = 6 month mark; 3 = final.

**Table 5:** Leg Power, TUG, 6MWT, and 4SST.

Participant	Leg power 1	Leg power 2	Change	TUG 1	TUG 2	Change	6MWT 1	6MWT 2	Change	4SST 1	4SST2	Change
FP002	17.09	18.60	**1.51**	9.21	8.72	−**0.49**	438.5	430.3	−**8.2**	6.85	5.94	−**0.91**
FP003	9.50	9.73	**0.23**	10.15	7.93	−**2.22**	339	400.2	**61.2**	5.41	4.65	−**0.76**
FP004	18.00	18.71	**0.71**	8.91	8.37	−**0.54**	436	394.2	−**41.8**	4.29	4.08	−**0.22**
FP006	13.19	11.86	−**1.33**	8.61	8.29	−**0.32**	372	372	**0**	6.02	5.14	−**0.88**
FP007	18.89	16.37	−**2.52**	7.39	7.99	**0.60**	420	462.5	**42.5**	4.27	4.23	−**0.05**
FP008	13.56	13.05	−**0.51**	9.33	9.83	**0.51**	428	417	−**11**	5.02	4.92	−**0.09**
FP009	8.97	8.09	−**0.88**	11.20	10.88	−**0.32**	279	264.8	−**14.2**	6.83	5.37	−**1.46**
FP010	14.78	14.97	**0.19**	8.28	8.20	−**0.09**	420	434	**14**	5.26	5.19	−**0.07**
FP011	16.24	19.56	**3.32**	8.08	8.19	**0.11**	366	363.1	−**2.9**	5.91	4.42	−**1.49**
FP012	12.95	17.10	**4.15**	9.82	10.08	**0.26**	372	413.5	**41.5**	7.49	4.46	−**3.04**
FP013	9.82	17.22	**7.40**	8.50	6.02	−**2.48**	394	439	**45**	6.22	3.96	−**2.27**
FP017	26.15	20.04	−**6.11**	8.07	7.36	−**0.71**	432.3	442.4	**10.1**	6.20	5.21	−**0.99**
FP018	9.47	10.37	**0.90**	7.16	7.52	**0.36**	396	466.25	**70.25**	7.10	4.25	−**2.86**
FP019	18.61	19.68	**1.07**	7.54	6.19	−**1.35**	412	442	**30**	3.44	3.06	−**0.38**
FP023	13.77	14.66	**0.89**	8.50	8.61	**0.11**	420	438	**18**	5.24	4.66	−**0.59**

Leg power = [(weight ∗ height of stairs (m))/time (sec)] (positive values indicate improvement).

TUG: timed up-and-Go test (negative values indicate improvement).

6MWT: 6-minute walk test (positive values indicate improvement).

4SST: four square step test (negative values indicate improvement).

1 = baseline; 2 = final.

**Table 6:** Spatiotemporal and kinematic data baseline and final intervention.

Spatiotemporal and kinematic variables	1.2 m·s^−1^			Self-selected			1.4 m·s^−1^		
	Mean	SD	*P*	Mean	SD	*P*	Mean	SD	*P*
Walking speed (m·s^−1^)									
Baseline	1.18	0.04	**0.00**	1.27	0.18	0.14	1.37	0.05	0.19
Final	1.20	0.04		1.30	0.18		1.38	0.04	
Cadence (steps/min)									
Baseline	56.99	3.74	0.07	60.00	4.85	0.33	60.71	3.09	0.07
Final	57.79	3.75		60.47	3.49		61.47	3.74	
Step length (cm)									
Baseline	63.2	6.9	0.01	62.5	3.7	0.53	67.9	3.5	0.05
Final	64.6	7.3		62.7	3.7		68.2	4.0	
Stride length (m)									
Baseline	1.25	0.08	0.42	1.27	0.13	0.36	1.35	0.08	0.59
Final	1.26	0.08		1.28	0.17		1.35	0.08	
Step width (m)									
Baseline	0.08	0.04	0.65	0.09	0.04	0.54	0.09	0.04	0.13
Final	0.08	0.04		0.08	0.04		0.08	0.04	
Stance (% of gait cycle)									
Baseline	61.91	2.81	0.46	61.45	2.41	0.11	60.54	2.07	0.89
Final	61.70	2.17		61.02	2.19		60.58	1.86	
Single stance (% of gait cycle)									
Baseline	38.27	3.76	0.39	38.53	2.81	0.17	39.30	2.83	0.81
Final	38.58	2.29		38.96	2.56		39.37	2.21	

Anterior pelvic tilt (°)									
Baseline	14.25	6.05	**<0.01**	14.78	5.83	**<0.01**	14.84	6.42	**<0.01**
Final	11.95	5.79		12.01	5.97		12.18	6.17	
Hip extension (°)									
Baseline	−9.93	8.30	0.04	−9.68	8.02	<0.01*	−10.42	8.58	0.02
Final	−11.98	8.81		−12.46	9.04		−12.96	9.58	
Hip flexion (°)									
Baseline	33.18	7.83	0.07	33.50	7.81	0.11	35.05	8.76	0.02
Final	31.63	6.94		32.10	7.10		32.76	7.31	
Ankle plantar flexion (°)									
Baseline	−11.24	4.86	0.24	−10.84	4.87	0.20	−12.92	5.28	0.86
Final	−11.89	4.76		−11.57	4.93		−13.04	5.39	
Knee extension at heel contact (°)									
Baseline	5.05	5.01	**<0.01**	5.29	4.53	**<0.01**	6.63	4.34	0.04
Final	7.06	5.19		7.34	5.01		7.81	5.20	
Knee flexion at load reception (°)									
Baseline	19.75	15.08	0.18	20.68	14.72	0.22	24.57	16.24	0.92
Final	22.07	14.59		22.71	13.49		24.39	13.61	
Knee extension at late stance (°)									
Baseline	1.20	6.31	0.08	1.27	6.38	0.94	0.81	6.87	0.09
Final	2.56	6.94		1.33	7.07		2.29	7.34	
Knee flexion at swing (°)									
Baseline	56.21	4.52	0.01	56.26	4.65	<0.01*	58.68	4.45	0.73
Final	57.66	5.46		57.90	5.44		58.87	4.82	

**Table 7:** Kinetic data baseline and final intervention.

Joint powers (W/kg)	1.2 m·s^−1^			Self-selected			1.4 m·s^−1^		
	Mean	SD	*P*	Mean	SD	*P*	Mean	SD	*P*
Ankle power absorption at heel off (A1)									
Baseline	−0.83	0.22	0.41	−0.87	0.24	0.27	−0.86	0.26	0.60
Final	−0.81	0.26		−0.91	0.32		−0.84	0.25	
Ankle power generation at late stance (A2)									
Baseline	3.69	0.58	0.50	3.95	1.00	0.13	4.22	0.73	0.36
Final	3.73	0.57		4.12	0.97		4.30	0.76	
Hip extensor power generation at stance (H1)									
Baseline	0.46	0.27	0.73	0.51	0.32	0.05	0.60	0.34	0.50
Final	0.44	0.28		0.58	0.34		0.57	0.33	
Hip flexor power absorption at late stance (H2)									
Baseline	−0.84	0.42	0.61	−0.94	0.52	0.40	−0.99	0.51	0.43
Final	−0.86	0.41		−0.99	0.45		−1.04	0.45	
Hip flexor power generation at toe-off (H3)									
Baseline	1.60	0.47	0.42	1.77	0.51	0.32	1.95	0.43	0.97
Final	1.56	0.42		1.83	0.55		1.95	0.40	
Knee flexor power generation at heel contact (K0)									
Baseline	0.78	0.51	0.99	0.93	0.58	0.10	1.01	0.60	0.16
Final	0.78	0.44		1.04	0.63		1.12	0.66	
Knee extensor absorption power at initial stance (K1)									
Baseline	−0.67	0.42	0.02	−0.88	0.56	0.04	−1.12	0.72	0.17
Final	−0.80	0.46		−1.01	0.52		−1.23	0.61	
Knee extensor power generation at mid stance (K2)									
Baseline	0.44	0.27	0.24	0.57	0.38	0.58	0.69	0.43	0.94
Final	0.48	0.31		0.59	0.33		0.69	0.34	
Knee extensor power generation at late stance (K3)									
Baseline	0.32	0.24	0.22	0.38	0.33	0.37	0.39	0.32	0.12
Final	0.28	0.25		0.35	0.31		0.33	0.25	
Knee extensors power absorption at initial swing (K4)									
Baseline	−1.25	0.42	0.22	−1.42	0.46	0.77	−1.52	0.37	0.76
Final	−1.20	0.35		−1.44	0.49		−1.51	0.40	
Knee flexors power absorption at final swing (K5)									
Baseline	−1.36	0.35	0.01	−1.57	0.55	<0.01*	−1.67	0.45	**<0.01**
Final	−1.47	0.35		−1.74	0.59		−1.86	0.46	
