# Supplementary material for: Moving with Ease: Feldenkrais Method Classes for People with Osteoarthritis
Source: Evid Based Complement Alternat Med. 2013 Sep 3;2013:479142. doi: 10.1155/2013/479142 (PMC3776373; doi:10.1155/2013/479142)
Supplement: Supplementary file 1 — The Supplementary Material includes a list of all the Feldenkrais Awareness Through Movement lessons undertaken by participants in this study, together with a description of the lessons. [file 479142.f1.docx]

**Appendix**

**Moving With Ease - Feldenkrais Method lessons for people with Osteoarthritis**

**Segment 1 – Description of lessons**

| **Number** | **Lesson Name** | **Lesson Description** |
| --- | --- | --- |
| 1 | Sliding Arms and Legs | Lying on the side, arms on the floor, sliding hand away from body then knee away from body with variations. |
| 2 | Legs like a Frog #1 | Lying on your back, legs long, allowing knee to open to side and drawing up first one leg then the other to the point of lifting off and bringing knees over stomach. |
| 3 | Learning to Fold | Lying on your back, one hand holding knee, the other lifting head, bringing elbow towards knee, with variations to activate flexors. |
| 4 | Arm Circles | Side lying, taking upper arm behind then in circles |
| 5 | Improving Flexors and Extensors | Lying on your back, knees crossed, allowing knees to fall to the side then having hands together with arms straight and taking hands opposite to knees to develop counter-rotation in spine. |
| 6 | Legs like a Frog #2 | Lying on your back, continuation of lesson 2. Developing the idea of using the back muscles to assist bringing the knees over the stomach. |
| 7 | Hand to Face Rolling | Side lying - with hand to face in various position, rotating upper body |
| 8 | Learning Comfort in the Baby Position | In the baby position - sliding hands on lower leg, then holding foot and lifting foot in various combinations. |
| 9 | The Pelvic Clock | Circular movements of the pelvis – Lying on your back and sitting. |
| 10 | Feet & Toes | Differentiating the movement of the feet & toes and fingers & hands. |
| 11 | Iron Bar Lesson | Lying on your back, hands raised to ceiling with fingers interlaced, tilting hands side to side. |
| 12 | Lying to Sitting | Lying on your back to side sitting. |
| 13 | Side Sitting Rotation | Side sitting, hand to face , improving rotation. |
| 14 | Side Lying Rotation | Lying on one side, opening chest to rotate arm behind. |
| 15 | Finding Stability through the Ankle | Lying on your back: one leg standing, pushing through foot to roll the pelvis, then lifting inside an outside of foot to mobilize ankle. |
| 16 | Head Circles | Sitting – Making circles with the head. |
| 17 | Rocking the Pelvis | Lying on your back, tilting the pelvis forward and backyards to establish the connection between the head and the pelvis. |
| 18 | Rolling Side to Side | Lying on your side, beginning with lifting the leg, then incorporating rotating the arm behind, developing a rolling from side to side. |
| 19 | Hip & Shoulder Integration | Side lying, developing circular movements of the hip and shoulder. |
| 20 | Lengthening the Back | Lying on your back, one leg raised, sliding hand along leg in various combinations. Lesson develops to the point where the foot is held with both hands, raised towards the ceiling and making circular movements. |

**Segment 2 – Description of lessons**

|  | **Lesson Name** | **Lesson Description** |
| --- | --- | --- |
| 1 | Integrating the Ankles and the Pelvis | Lying on your back, lifting the front of the foot then the heel, involving pelvic tilt in movement of ankles. |
| 2 | Getting to know your Hip Joints | Lying on your back, bringing knee to chest while at the same time arching your back and tilting pelvis in an opposing movement. Gives a clear sense of the Hip Joints. |
| 3 | Circling the Ankles | Lying on your back, knee bent, beginning with knee inward and outward developing into circles of the toes with heel on the ground. |
| 4 | Sitting on Heels - Part 1 | On hands and knees, beginning the exploration of sitting on heels. However, the lesson does not yet get to the point of actually sitting on heels. |
| 5 | Holding the Foot and Straightening the leg - Part 1 | Holding the foot (or the big toe, or the ankle) and straightening the leg; firstly on the back, then rolling to the side. |
| 6 | Hugging | Lying on your back, arms crossed and moving in a sawing motion. |
| 7 | Improving the Bending of the Knees | Lying on your back, bringing knee to chest, elbow to knee and incorporating the bending of the ankle. |
| 8 | Diagonals – Part 1 | Lying on your back: one leg standing, pushing foot into floor to roll, adding in reaching with same arm as leg pushes, then reaching with opposing arm. |
| 9 | Rolling Fists | Lying on your back – arms out to sides, rolling the fists with various combinations of pelvic tilting and lifting head. |
| 10 | Finding Stability in Standing | Standing behind a chair, bending in all joints. Develops tilting of pelvis to assist bending in knees and ankles. |
| 11 | Sitting on the Heels – Part 2 | On hands and knees, develops further the movement of sitting back on the heels. Develops into a movement of going from hands and knees to side sitting. |
| 12 | Sitting on a Chair to Standing | Explores how to improve the efficiency of going from sitting in a chair to standing. |
| 13 | The Flying Lesson | Lying on your stomach: Arms are outstretched, beginning by lifting fingers, develops into lifting both arms. |
| 14 | Holding the Foot and Straightening the leg – Part 2 | Very similar to Lesson 5. Doing it a second time to develop ease in doing the movements. |
| 15 | Learning to Side Sit more easily | Side sitting, sliding hand away from the pelvis whilst looking up then down. |
| 16 | Diagonals – Part 2 (reaching) | Lying on your back, one leg standing, pushing with the foot to engage the diagonal arm in reaching. |
| 17 | Learning to Sit Crossed Leg more easily | Lying on your back, one foot standing on the other ankle, lifting inside and outside of foot. |
| 18 | Feet against the Wall | Lying on your back with your feet up against a wall, pushing with feet. Various movements of the foot improve the ability to push. |
| 19 | Sitting on Heels – Part 3 | On hands and knees, flexing and extending feet to improve sitting backwards towards heels. |
| 20 | A Flexible Back | Sitting, lying and on elbows, feet together, tilting knees side to side. Improves hip mobility. |

**Segment 3 – Description of lessons**

|  | **Lesson Name** | **Lesson Description** |
| --- | --- | --- |
| 1 | Twisted | Lying on your side, with the upper body facing ceiling, alternating reaching with both arms. |
| 2 | Foot and Ankle Movements | Lying on your back, exploration on movements of the foot, toe and ankle. Explorations continue in standing. |
| 3 | Arms in the shape of an X | Starting lying on your back, arms and legs extended to make an X shape, reaching along the diagonals. Exploration continues lying on your stomach. |
| 4 | Rolling like a Baby | Starting lying on your back, elbows and knees above trunk, developing a reaching movement to right onto right side then left side. |
| 5 | Painting with the Soles of the Feet | Lying on your back, sliding the foot away and right to left while maintaining the contact with the floor by the sole of the foot. |
| 6 | Freeing the Neck | Lying on your stomach, developing a movement of turning and looking over one shoulder then another. Improves the body’s ability to extend. |
| 7 | From Side Sitting to Kneeling | Starting in side sitting, learning to move forward over front leg into a kneeling position. |
| 8A | Knees Bend Knees | Standing, legs crossed over, using the back knee to encourage the front knee to bend more easily |
| 8B | Neck Relief | Sitting, some simple movements to release neck and shoulder tension. |
| 9 | Side Bending with a Twist | Lying on your back with knees crossed over and then falling to one side, taking head side to side in a lateral movement, encouraging ribs to soften. |
| 10 | Walking | Exploration of the movements of the shoulder in relation to the movement of the foot in walking. |
| 11 | Arms Like a Candelabra | Lying on your back, arms out to the side with elbows bent at right angles, taking forearms down and up. |
| 12 | The Head in Walking | Exploration of the movements of the head in relation to the movement of the foot in walking. |
| 13 | Freeing the Hip Joints in Sitting | Sitting in a chair, ankle on other knee, lifting the leg and reaching through the gap to improve hip joint flexibility. |
| 14A | Knees Straighten Knees | Standing, legs crossed over, using the front knee to encourage the back knee to straighten more easily |
| 14B | Twisting in a Chair | Sitting in a chair, holding onto back of chair with one hand, rotating upper body with variations in the movement of the head, to improve turning. |
| 15 | A Morning Routine | A series of movements from different Awareness Through Movement lessons designed as a routine for morning exercise. |
| 16 | Walking Backwards | A lesson done in standing and walking backwards to encourage lengthening as you walk. |
| 17 | Flexible Knees | On hands and knees, learning to improve sitting back on heels. |
| 18 | Holding the Foot and Straightening the Leg Part 3 | Holding the foot and straightening the leg; firstly on the back, then rolling to the side, then taking the knee either side of the arm. |
| 19 | Modigliani Rolling | Lying on your back, pushing through the diagonal foot to reach. Based on the Modigliani model. |
| 20 | Learning to Fold – Elbow to knee combinations | A rediscovery of Segment 1, lesson 3. Lying on your back, one hand holding knee, the other lifting head, bringing elbow towards knee, with variations to activate flexors. |
